# Supplementary material for: The influence of cross-border mobility on the COVID-19 epidemic in Nordic countries
Source: PLoS Comput Biol. 2024 Jun 12;20(6):e1012182. doi: 10.1371/journal.pcbi.1012182 (PMC11198903; doi:10.1371/journal.pcbi.1012182)
Supplement: S3 Appendix — (PDF) [file pcbi.1012182.s003.pdf]

The influence of cross-border mobility on the COVID-19 epidemic in Nordic countries
S3 Appendix: Additional Results

June 9, 2024

Mikhail Shubin[1 *], Hilde Kjelgaard Brustad[2], Jørgen Eriksson Midtbø[3], Felix Günther[4], Laura Alessandretti[5], Tapio Ala-Nissila[6, 7], Gianpaolo Scalia Tomba[4, 8], Mikko Kivelä[9], Louis Yat Hin Chan[3], Lasse Leskelä[1]

1 Department of Mathematics and Systems Analysis, Aalto University, Espoo, Finland
2 Oslo Center for Biostatistics and Epidemiology, Oslo University Hospital, Oslo, Norway
3 Department of Method Development and Analytics, Norwegian Institute of Public Health, Oslo, Norway
4 Department of Mathematics, Stockholm University, Stockholm, Sweden
5 DTU Compute, Technical University of Denmark, Copenhagen, Denmark
6 Quantum Technology Finland Center of Excellence, Department of Applied Physics, Aalto University, Espoo, Finland
7 Interdisciplinary Centre for Mathematical Modelling and Department of Mathematical Sciences, Loughborough University, Loughborough, United Kingdom
8 Department of Mathematics, University of Rome Tor Vergata, Rome, Italy
9 Department of Computer Science, Aalto University, Espoo, Finland

* mikhail.shubin@aalto.fi

In this appendix, we present some additional results complementing those in the main text.

1 Model estimates

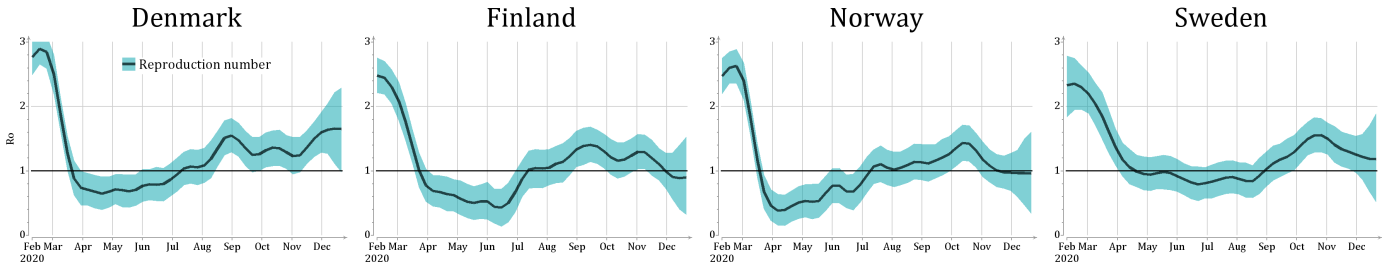

Figure A: Estimates of the reproduction number $\mathcal{R}_{t,x}$. Posterior mean and 90% credible intervals.

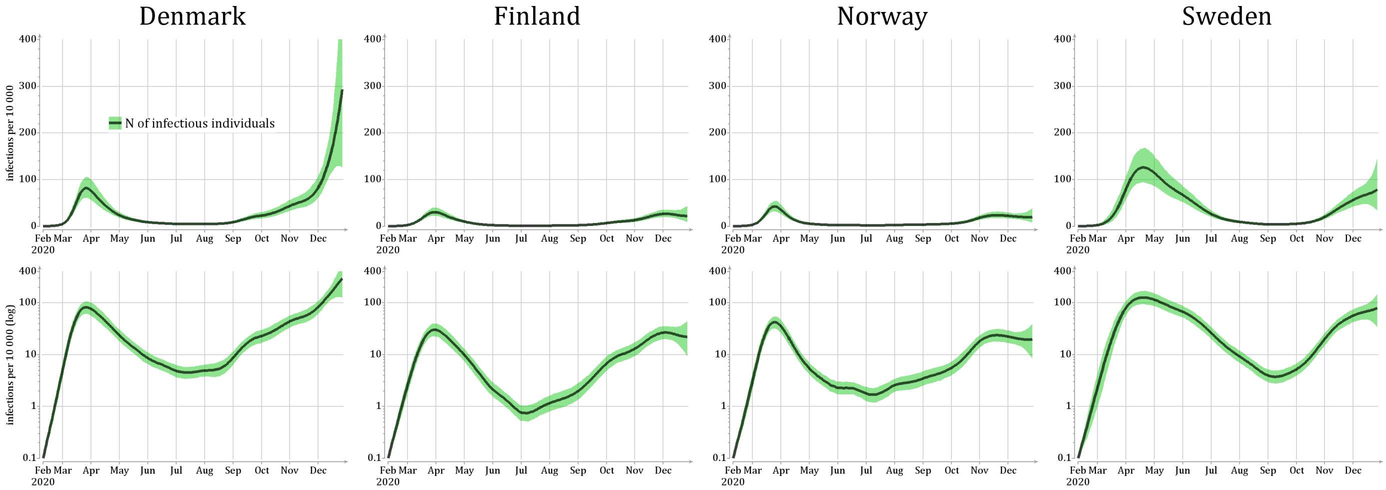

Figure B: Estimated numbers of infectious individuals $I_{t,x}$, in linear and log scales. Posterior mean and 90% credible intervals.

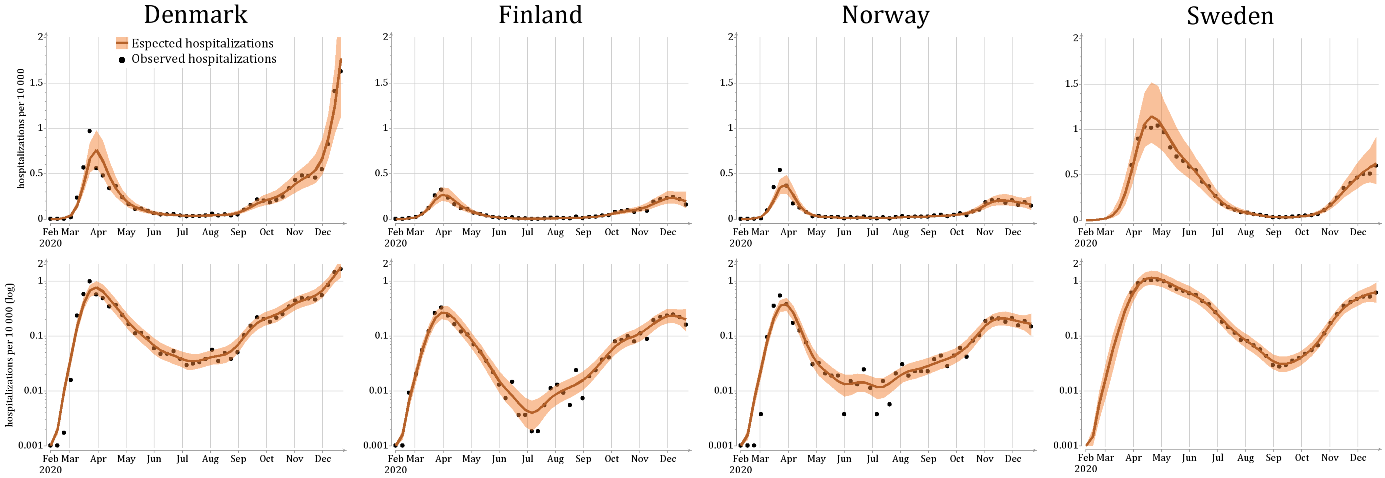

Figure C: The expected number of hospitalizations per week $E_{w,x}$ and the corresponding observed numbers observed numbers $H_{w,x}$ in linear and log scales. Posterior mean and 90% credible intervals, dots present the observed data.

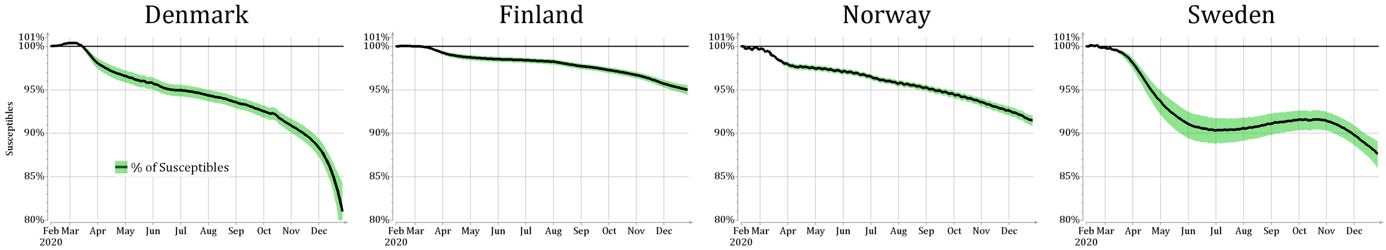

Figure D: Estimated portion of Susceptibles in the population $S_{t,x}/N_{0,x}$. Posterior mean and 90% credible intervals. Note that y-axis does not include zero.

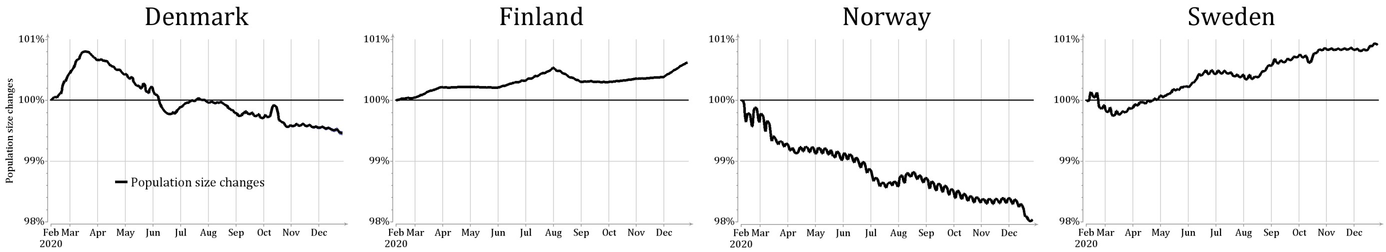

Figure E: Changes in the population size in countries due to mobility $N_{t,x}/N_{0,x}$, relative to the starting population size. Posterior mean and 90% credible intervals. Note that y-axis does not include zero.

2 Primary effect estimations

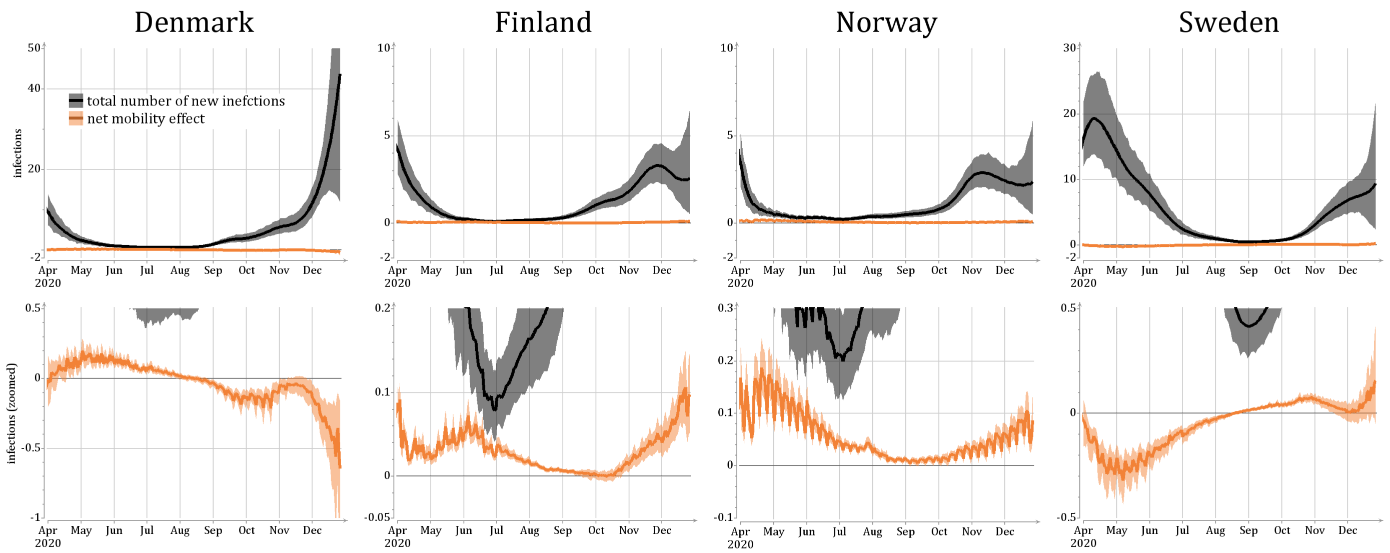

Figure F: Estimates of the total number of new infections per day $i_{t,x}^{\text{total new}}$ local and the net mobility effect $Q_{t,x}$, shown at different linear scales. Posterior mean and 90% credible intervals. The upper row shows the $i_{t,x}^{\text{total new}}$ on a larger scale, while the lower row zooms in to focus on $Q_{t,x}$.

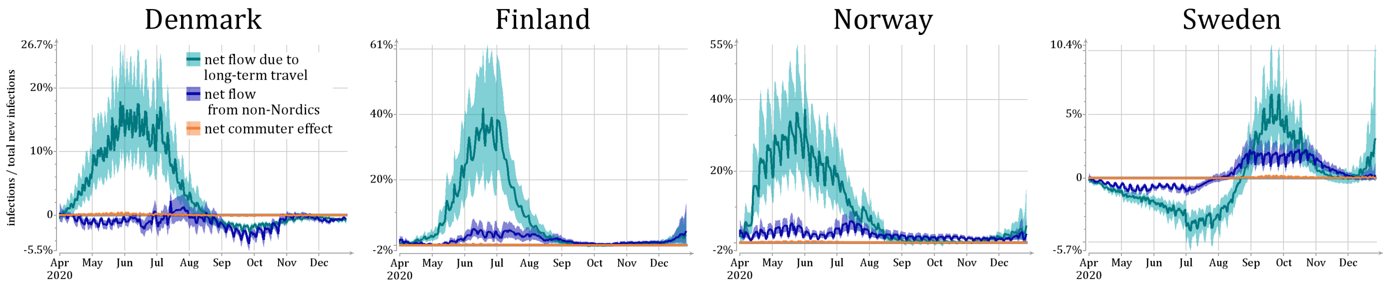

Figure G: Estimates of new non-local infection by source, divided by the total number of new infection: $Q_{t,x}^{\text{long term}}/i_{t,x}^{\text{total new}}$, $Q_{t,x}^{\text{non-Nordic}}/i_{t,x}^{\text{total new}}$ and $Q_{t,x}^{\text{commuters}}/i_{t,x}^{\text{total new}}$. Posterior mean and 90% credible intervals. See Section 3.1.1 for definitions. Note that Figure 4 in the main text shows the same quantities $Q^{\text{long term}}$, $Q_{t,x}^{\text{non-Nordic}}$ and $Q^{\text{commuters}}$ in absolute scale

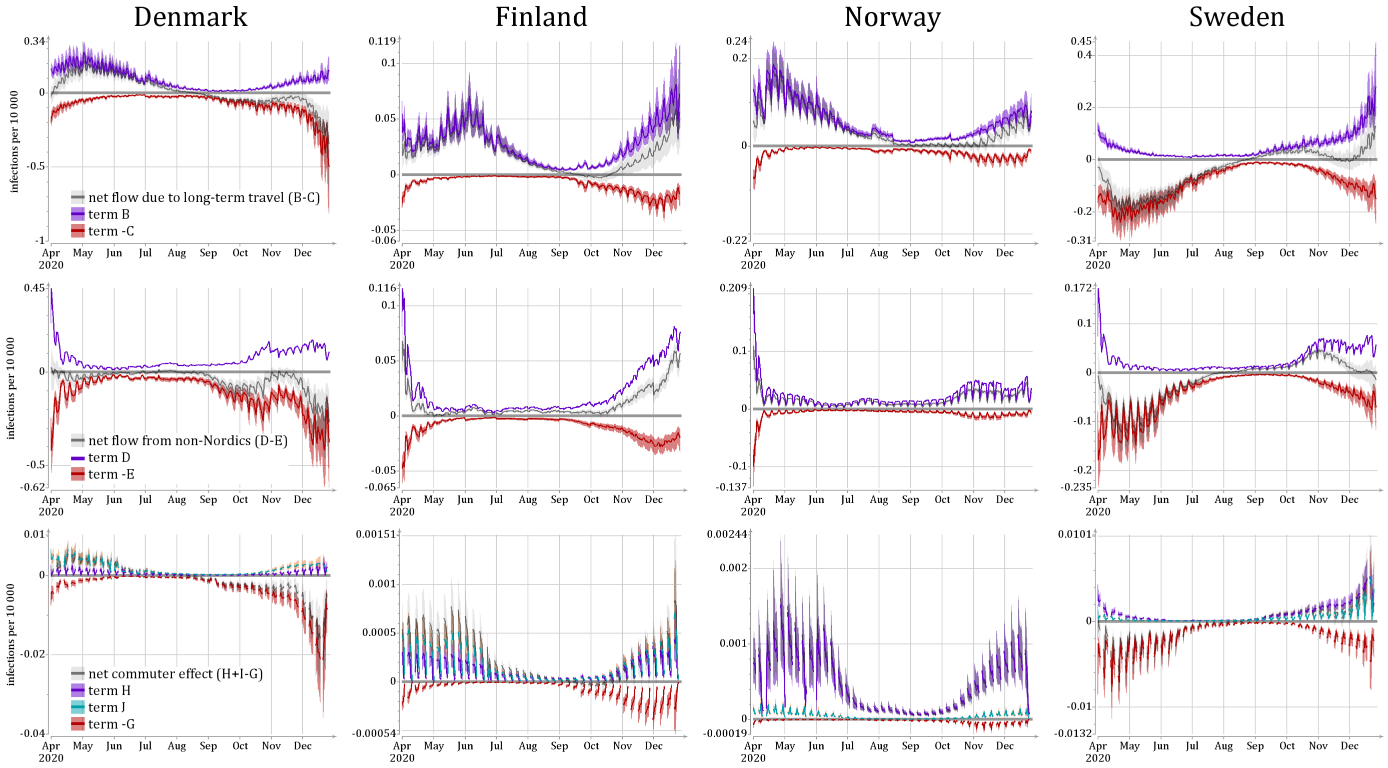

Figure H: Estimates of the mobility effects, split into different contributing factors. Posterior mean and 90% credible intervals.
Term B: infected from other Nordics arriving to country x.
Term C: departing from country x to other Nordic countries.
Term D: inflow to country x from outside of Nordics.
Term E: departing from country x to outside of Nordics.
Term G: reduction in local infections due to commuters leaving.
Term H: infections caused in country x by commuters arriving from other Nordics.
Term J: commuter from country x, infected in other countries and returning back.
See Section 3.1.1, in particular equations (11) and (13) for detailed definitions.

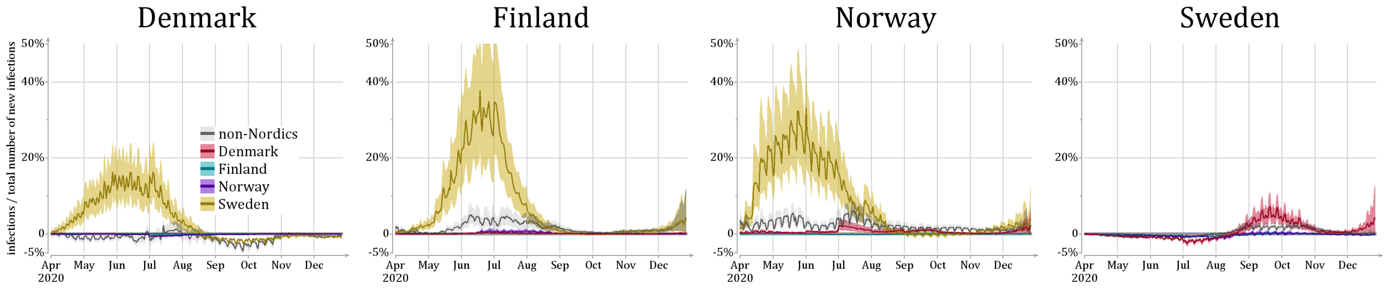

Figure I: Estimates of non-local infections by country per day, divided by the total number of new infection. Lines show posterior mean and colored areas show 90% posterior intervals. Note that Figure 5 in the main text shows the same quantities in absolute scale.

3 Secondary effect estimations

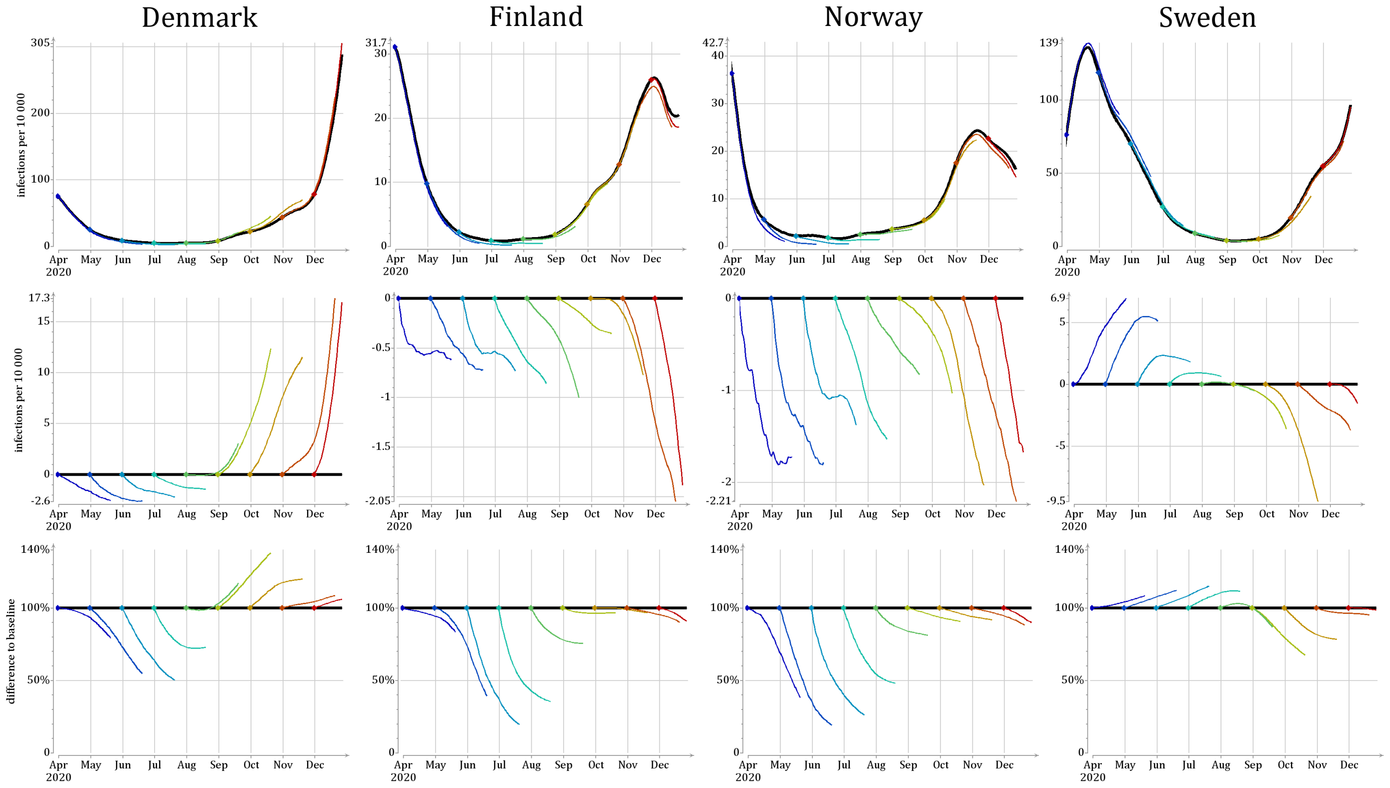

Figure J: Alternative representation of the counterfactual analysis, where cross-country mobility is set to zero. Top row: black line shows the mean number of infections $E(I_{t,x})$ in the baseline scenario. Colored lines presents the number of infections in counterfactual scenarios $E_c(I_{t,x})$, during the 50 day interval starting with the implementation of restrictions. Dots mark the start of the restriction. Mid row: same values, shown as a difference between counterfactual and baseline scenario $E_c(I_{t,x}) - E(I_{t,x})$. Bottom row: same values, divided by baseline scenario $[E_c(I_{t,x}) - E(I_{t,x})]/E(I_{t,x})$. Note that Figure 8 in the main text shows the same quantities $E(I_{t,x})$ and $E_c(I_{t,x})$ in log scale.

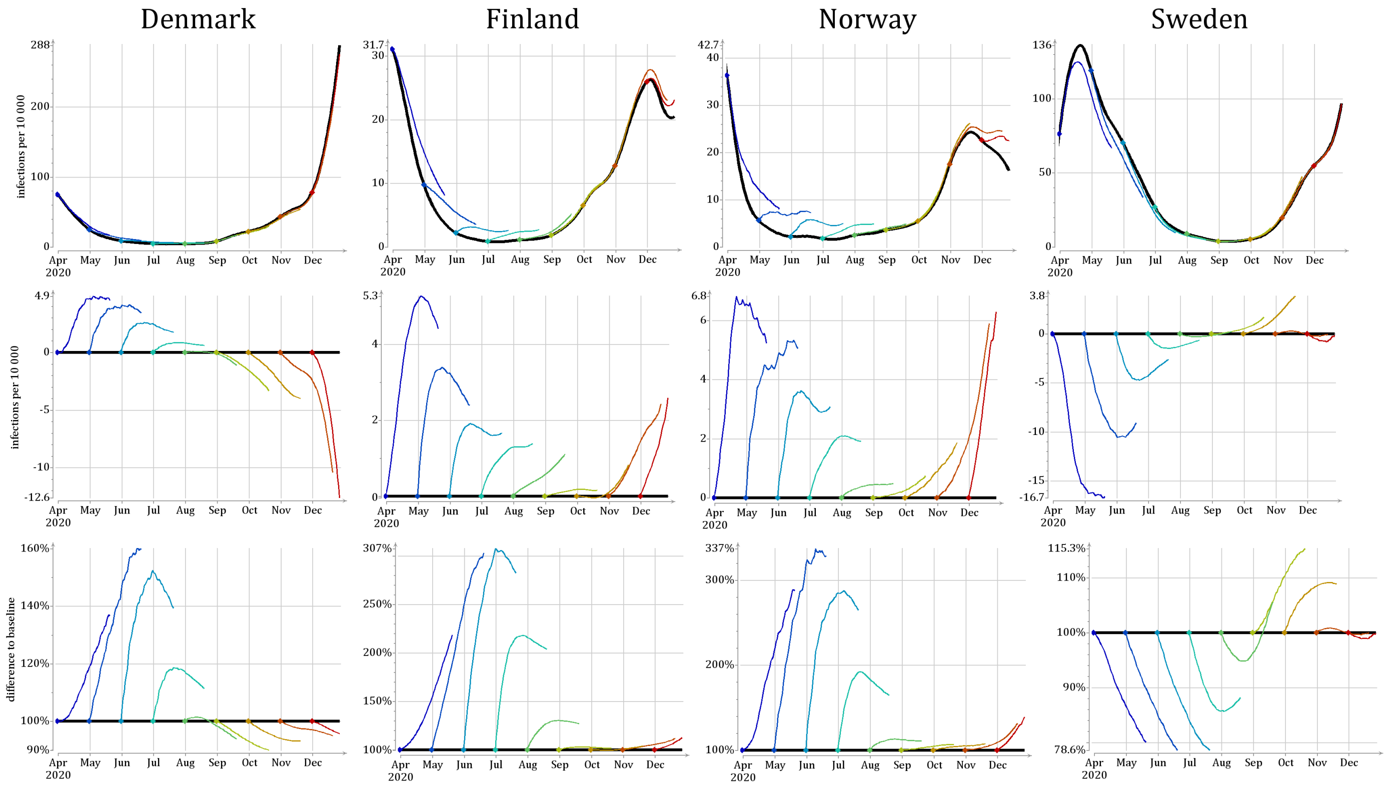

Figure K: Alternative representation of the counterfactual analysis, where mobility returns to the pre-pandemic levels. Top row: black line shows the mean number of infections $E(I_{t,x})$ in the baseline scenario. Colored lines presents the number of infections in counterfactual scenarios $E_c(I_{t,x})$, during the 50 day interval starting with the implementation of restrictions. Dots mark the start of the restriction. Mid row: same values, shown as a difference between counterfactual and baseline scenario $E_c(I_{t,x}) - E(I_{t,x})$. Bottom row: same values, divided by baseline scenario $[E_c(I_{t,x}) - E(I_{t,x})]/E(I_{t,x})$. Note that Fig 9 in the main text shows the same quantities $E(I_{t,x})$ and $E_c(I_{t,x})$ in log scale.

4 Posterior correlation of the parameters

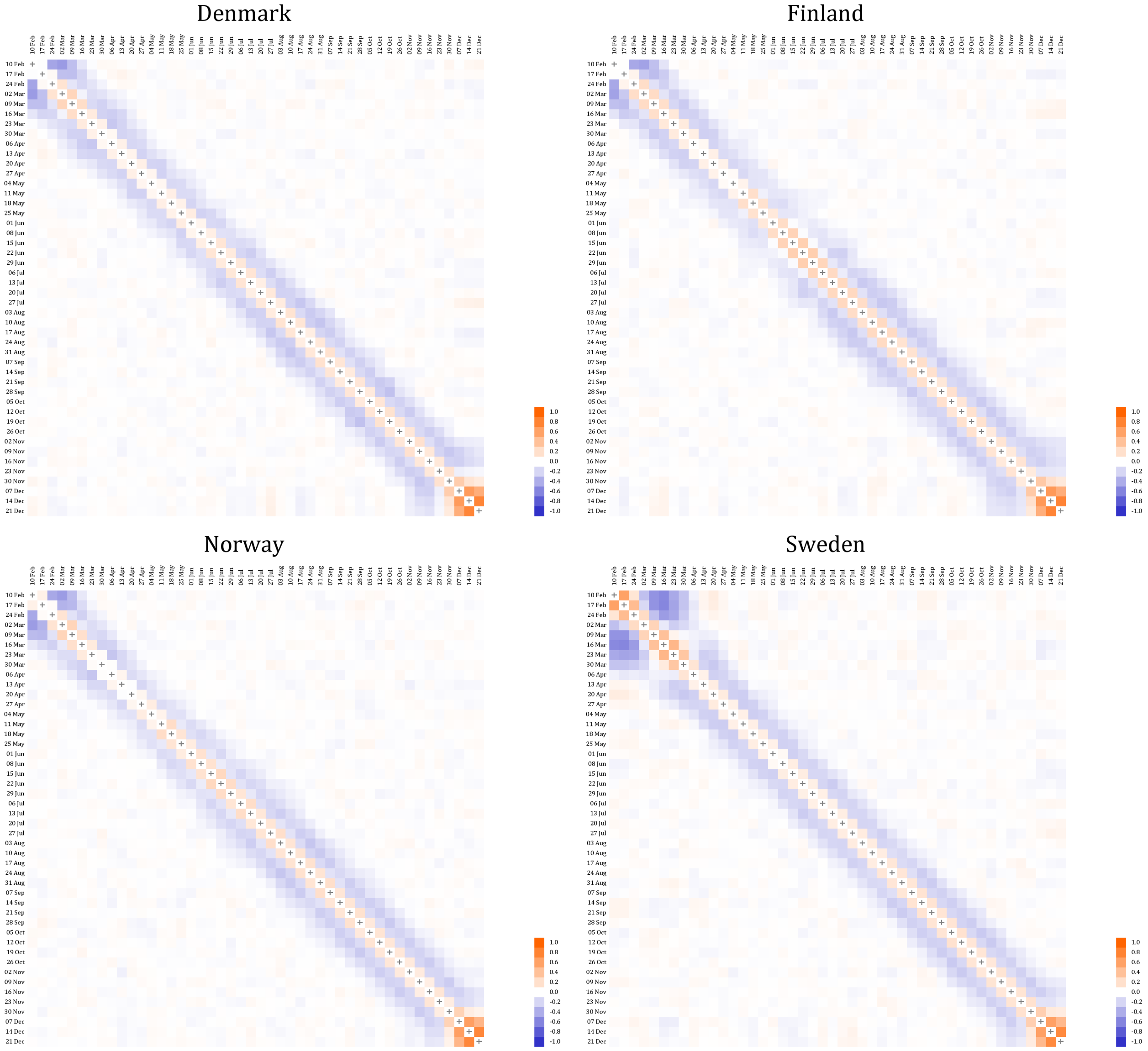

Figure L: Posterior correlation between values of $\mathcal{R}_{tx}$ in each country. We see that for the majority of the values, sequential values (time difference one week) are slightly positively correlated; values 2-5 weeks apart are slightly negatively correlated and the rest are uncorrelated. Values at the beginning and end of the modelled period are more strongly correlated (both positively and negatively).

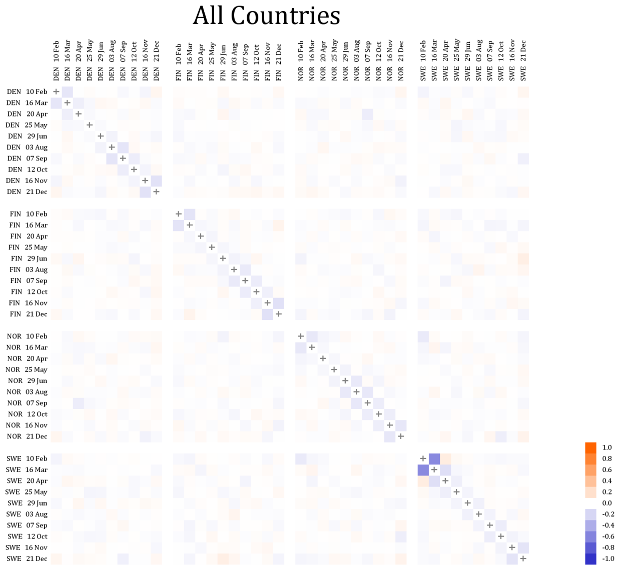

Figure M: Posterior correlation between selected values of $\mathcal{R}_t$ for all countries. We see that the values of $\mathcal{R}_{tx}$ are not correlated between different countries.
