# Supplementary material for: The influence of cross-border mobility on the COVID-19 epidemic in Nordic countries
Source: PLoS Comput Biol. 2024 Jun 12;20(6):e1012182. doi: 10.1371/journal.pcbi.1012182 (PMC11198903; doi:10.1371/journal.pcbi.1012182)
Supplement: Attachment — Submitted filename: replies_with_appedix.pdf [file pcbi.1012182.s004.pdf]

March 21, 2024

PLOS Computational Biology
Manuscript number PCOMPBIOL-D-23-01783
Original manuscript submitted on 2 Nov 2023
Revision requested on 6 Feb 2024

Dear Editors and Associate Editors,

We thank you for your time in evaluating our submission. In particular, we are grateful for the helpful comments of the anonymous reviewers. Based on the feedback, we have carefully revised our manuscript. A point-to-point response and the essential changes are included below. We hope that this revised manuscript properly addresses the points raised by the reviewers and meets the high quality standards of articles published in PLOS Computational Biology.

Sincerely,

Dr Mikhail Shubin (also on behalf the other authors)

Response to Reviewer #1

We thank Reviewer #1 for a thorough reading of our manuscript and providing sharp comments and remarks which have helped to improve the text.

Major comments

R1.1. During the first year of the COVID-19 pandemic, there were many indications that super-spreading events were to some extent driving the pandemic (see 33139561[pmid]). How is this reflected by the model (if at all) and what impact can super-spreading events have on travel between countries? (i.e. can travel by boats, trains or planes function as super-spreading events? (see 35779143[pmid], 32726405[pmid]) How can such super-spreading events go on to drive secondary infections in the host countries? (This is also discussed somewhat in the referenced Creswell et al. article). It is not expected that the authors redo their analyses of course but a couple of sentences describing super-spreading and how this might be challenging to account for with standard SIR models would be helpful.

A1.1 This question can be split into two parts: 1) can super-spreading events be accounted for in our model (and thus, does our model fit the data correctly) and 2) is super-spreading more likely for infectious individuals making a cross-border travel?

1) In the paper, we are using a deterministic SIR model, which is based on average numbers of secondary infections. Super-spreading is usually seen as a random event which makes the distribution of the number of secondary infections heavy-tailed. When the number of infectious individuals in the population is high, the effects from super-spreading would be averaged out; SIR models have no problems with simulating these dynamics. However, when the number of infectious individuals is low (for instance, we estimated that in Finland in June there were less than 1 infection per 10000), extreme fluctuations caused by super-spreading may not be well described by a deterministic SIR model. However, in our model we use a time-varying infectivity parameter, so the presence of superspreading events may be implicitly captured as a fluctuation of R_t.

2) Is super-spreading more likely for infectious individuals making cross-border travel? In our paper we don't compare travelling behaviour to non-travelling behaviour, but cross-border travel to staying within the country (which includes within-country travel). I.e. in our study, transmission occurring during local plane/train travel is considered local. Note that for the countries with elongated shapes (Norway, Sweden and Finland) local travel can be longer than cross-country one.

We added the following text to the paper:

To Table 1 describing the R_t parameter: This number aggregates the factors influencing the spread of infection: interventions, changes in population behaviour, seasonal effects, super-spreading outbreaks.

To the second-to-last paragraph in the Discussion: There are other important differences between the real epidemic and an SIR model. Super-spreading is suggested as a driving factor for the pandemic during the studied period. Super-spreading implies a heavy tailed distribution of secondary infections, while the deterministic SIR model assumes a constant number. In addition, assortative mixing has been suggested as a factor influencing the infection dynamics. In our model we use a time-varying reproduction number $_t$, which should be able to capture the fluctuations caused by super-spreading and assortative mixing. It should also aggregate the differences between countries, e.g. differences in climate and pandemic suppression policies.

R1.2. The ability to identify the true effect of border crossings on infectiousness is likely very difficult to assess as it is clearly challenging to separate local infections from imported. The values presented are therefore only projections assuming nothing but the reproductive number from the source country it seems. This should be highlighted as a premise for the study and reflected upon when the consequences and conclusions are presented, particularly for the counterfactual part. Traveling itself may introduce unidentified risks not accounted for and may not have been controlled for in the study. This should be pointed out in the discussion and recommendations section.

A1.2. We added the following text to the Discussion:
Leaving one country to visit another may change the number, duration and context of social contacts. In our model, we assumed that people crossing the border have the same infectivity and susceptibility as the local people in the visited country (including those doing local travel). Collecting quantitative data on travellers' behaviour abroad and estimating additional risks imposed by the travel itself would help in further planning of intervention measures.

R1.3. All countries considered are well-governed and although similar there are also some marked differences. While Finland and Norway are fairly similar in terms of both population size/density and geography (both countries also share borders with Russia), Sweden has a population almost double the size and thus lower population density. Denmark, on the other hand, is located on the European continent, away from Norway and Finland, and has an island-like structure with a markedly higher population density. As an EU member (as both Sweden and Finland also are), Denmark shares open borders with Germany in addition to Sweden. I miss more discussion on how these differences can impact the spread of COVID-19 internally in each country as a consequence of importations (See for instance 35229003[pmid]. It doesn't have to be much but it should be mentioned how these differences are reflected in their model. The model is the same for all countries but the countries inform the model differently from the available data, can something have been lost?

A1.3. For the purpose of our study, the difference between internal transmission inside different countries are summarized by the different R_t values.

In order to infer the causes behind the specific values of R_t (e.g. why in April 2020 the epidemic was growing in Sweden and declining in Finland, Norway and Denmark, and if Swedish relaxed attitude towards COVID have something to do with it) one would have to take the context of each country (including population density, age-specific transmission matrices, media awareness etc etc) into consideration. However, for the purpose of this study, we consider R_t as a product of different, possibly unknown, factors. Similarly, with the data at hand and at the description level of our model, the effects of geography and demographics are summarized by the estimated amounts of border crossings (eg see Figure 1).

We have added population sizes of the countries to the text.

Q1.4. What effect on the population does the length of immunity and the reproduction number in a country have on imported cases and their role in driving (or reducing) the pandemic? That is, if a fraction of the population is immune and immunity lasts for a long time (say 6-12 months) how does this influence the effects of border crossings on the evolution of the pandemic within a country? How can this differ from say shorter duration of immunity as witnessed by the more recent SARS-CoV-2 Omega strains?

R1.4. During the modelled period (year 2020), the fraction of infected individuals in the modelled countries was low, with a maximum of 20% infected population in Denmark (see Appendix S3 figure 4). Such a population could be considered almost totally susceptible for the purpose of spreading the infection, and thus decreasing the time spent immune would not

affect the results. Estimating the effect of cross-border mobility in the more complex situation, when each country has a complex dynamic immunity landscape, would indeed be interesting. However, this lies outside the scope of this study. Furthermore, decreased immunity with respect to COVID-19 has especially been seen with introduction of new variants. During the period studied here, this only happened partially towards the end of the period and the effect would have been absorbed by the adaptive R_t in our model.

Q1.5. What about a country with a higher population density like Denmark, how might imported infections spread there compared to countries with a lower population density like Norway and/or Finland? Is it at all possible to separate the effects from importation in a country like Sweden with high infection rates from a country like Denmark with higher density, or will these countries appear the same from the proposed model?

A1.5. As mentioned in the Discussion, the lack of specific quantitative data on behaviour (from an infectious potential point of view) of travellers has led to the assumption that they behave as the local population in terms of infectivity and this, in turn, is represented by the local R_t. This R_t aggregates the effects of population density, demographical structure and social behaviour on infectivity.

Q1.6. Not much is revealed regarding the impact of Russian cross-border traffic in Norway and Finland in the main text, although some results are presented in the S1 appendix. How could these border crossings affect spread of COVID-19 and is it expected to differ from within Nordic countries border crossings?

R1.6. According to Appendix 1, the amount of border crossing from Russian to/from Finland and Norway is much lower than those from other countries (eg Sweden), it drops even lower after the emergence of COVID and does not recover in 2020. This is why we believe that the infection exchange with Russia is negligible compared to e.g. the one with Sweden.

Minor comments

Is it Shubin Mikhail or Mikhail Shubin (see S1-S3 appendix authors).

Corrected.

In S1 appendix, 2. page 2. paragraph: make sure you indicate the "o" index in $D_{t,d \leftarrow o}^{long/short}$

Index "o" stands for the "other countries". This is now explicit in the text.

In S1 appendix, first paragraph, page 7: Note that the because…

Corrected.

It is not always completely apparent what the figures in S3 appendix designate, some more information in the figure legends would be appreciated.

We expanded the figure captions in Appendix S3.

The initial reproduction number is defined to be Normal with mean=2 and sd=0.5, this can some times be negative, which is nonsensical, why not use log-normal?

The law of the initial reproduction number is not exactly Normal, but a truncated Normal that is forced to take on positive values. We have clarified this detail in the Section *2.3.3 Parameter model and Bayesian parameter inference.*

In equation 13 the letters of the different terms end up with the same letter “I” that also designates infectiousness.

We renamed factor I into J.

Response to Reviewer #2

We thank Reviewer #2 for a thorough reading of our manuscript and providing sharp comments and remarks which have helped to improve the text.

Major comments

Q2.1. Section 3.1.2 requires a more detailed explanation of how M is estimated. I suggest spelling out each term in equations 16 and 17.

R2.1. We expanded the corresponding text around equations (16) and (17)

Q2.2. Similar to above, the final sentence in section 3.1.3, 'Prevalence of infection among long-term travellers from outside Nordic countries is provided in the data', is not entirely clear. Is this part of the data that was fed to the model and is it thus fixed? This is not the impression I got from reading the rest of the methods, but I could be entirely wrong.

R2.2. To estimate the number of non-Nordic infected travellers, we have to rely on the airplane traffic data, which are unfortunately private and cannot be shared. To handle the privacy concerns, we pre-aggregated the data and fitted it into the model as the daily number of infected individuals entering the country (parameter i^{inflow}). The algorithm for obtaining i^{inflow} is described in the last section of Appendix S1 (Estimated number of non-Nordic infected travellers entering the Nordic countries).
We added the equation for 'the prevalence of infection among long-term travellers from outside Nordic countries' to the Appendix S1; we also added a reference to this appendix to the text.

Q2.3. I appreciate the graphical depiction of the model (Fig 2). However, it would be very helpful to have an expression for what the likelihood function is here. There are many parameters and if the data are just the number of hospitalisations per day, then stating out the likelihood here would help put everything into context.

R2.3. The likelihood is given by equations 1-5 and 8-9. In particular, Equation 9 links the observed data to the expected number of hospitalizations, Equation 8 links the expected number of hospitalization to the number of infection individuals, and equations 1-5 describe the SIR model: i.e. what would the number of infection individuals be given a time-dependent reproduction number R_t.
We have updated the caption of Fig. 2 to indicate the three submodels: transmission model (blue), parameter model (red), observation model (green), and provided references for the relevant equations in the Figure itself.

Q2.4. In Fig 2 I can see that there are several fixed components in the model. Can the authors comment on their uncertainty? For example, whether the hospitalisation probability or its probability after t days of infection have associated uncertainties. In that case, what would be the effect of treating these as stochastic nodes? Presumably not much, but I think this point would help the reader understand the extent to which the model is realistic.

R2.4. The fixed parameters are unidentifiable given the data used in this study, and thus they need to be either fixed or given an informative prior. We decided on fixing the parameters for the sake of computational burden.
Let us now discuss how changing these parameters could affect the conclusion of our study.
1) Hospitalization probability (h_x): increasing (or decreasing) the hospitalization probability would decrease (or increase) the estimated number of active infections. However, since the number (and fractions) of imported infections would be all scaled in the same way, our conclusions would not change. Theoretically, changing h_x would also affect the estimates of R_t, as having

a lower [or higher] portion of removed individuals in the population would require lower [or higher] R_t to produce the same effective reproduction number. However, during the modelled period (year 2020) the fraction of infected individuals in the modelled countries was low, with a maximum of 20% infected population in Denmark (see Appendix S3 figure 4), meaning that R_t stayed close to R_y^{eff}, so changing h_x should not cause large changes.
2)The probability that hospitalisation happens d days after infection (p_d): This parameter is based on direct data from hospitals in the modelled country, so we are quite confident in it.
3) Recovery rate (gamma). Increasing [decreasing] the recovery rate would decrease [increase] the mean time spent infective.
4) Average fraction of time that a commuter from country s spends in country d during a daily trip (alpha). Since the effect of commuters was estimated to be very low, changing this parameter from 0.5 to 1 or 0 would not affect the conclusion.

Q2.5. The authors have commented on variations of their model due to age and regional stratification. However, there are other important factors, like those that break the SIR assumptions. I suggest adding a few sentences on whether these can have an impact here.

R2.5. We added the following text to the second-to-last paragraph in the discussion:
There are other important differences between the real epidemic and an SIR model. Super-spreading are suggested as a driving factor for the pandemic during the studied period. Super-spreading implies a heavy tailed distribution of secondary infection, while the SIR model assumes a constant number. In addition, assortative mixing was as suggested as a factor influencing the infection dynamics. In our model we use a time-varying reproduction number R_t, which should be able to capture the fluctuations caused by super-spreading and assortative mixing. It should also aggregate the differences between countries, e.g. differences climate and pandemic suppression policies.

Q2.6. There is substantial evidence that Sweden had the highest viral genetic diversity of the virus among Nordic countries in the early stages of the pandemic. This indicates that it acted as a net exporter of the virus. Of course, these dynamics probably changed rapidly, with local transmission playing an increasingly big role in each jurisdiction. In the light of this work, this can simply mean that the number of importations was not as important as the fact that they actually resulted in ongoing transmission in each country. Can the authors comment on how the genetic evidence stacks up and its implications?

R2.6. The genetic evidence agrees with our conclusion that Sweden was an exporter of infections to other Nordic countries. Moreover, genetic studies have the same general methodological problem as our paper. For example, assume that we know that 10 000 infections in country X originate from a single infectious tourist from country Y. Should we then conclude that this tourist caused 10 000 infections, or that it is only responsible for a few direct ones and the rest are caused by local transmission (and would have happened anyway, even if said tourist had not arrived)? This is more of a philosophical question, and it has to be answered regardless of whatever the knowledge of the transmission tree is based on mathematical modelling or direct genetic evidence.
In our paper we developed multiple metrics that can help to disentangle the cross-border transmission, and we conclude that there is no single correct one.

Minor comments

Q2.7. There are a few minor typological or grammatical errors that can be easily fixed with careful proofreading. E.g. line 243 'between by country' – remove the 'by'.

R2.7. Corrected.

Response to Reviewer #3

We thank Reviewer #3 for a thorough reading of our manuscript and providing sharp comments and remarks which have helped to improve the text.

Major comments

Q3.1. One aspect that the article does not address is the distinction between different COVID-19 virus variants. While the dominance of the Wuhan variants is evident for most of 2020, the emergence of the VOC Alpha variant in December is not sufficiently highlighted.

R3.1. Indeed, introduction of multiple VOCs increases the complexity of our subject. For this reason, we chose 2020 as our model year: it was dominated by a single strain of Covid.
While Alpha strain was detected in Nordic countries already in December 2020, it has reached dominance only in 2021. You can see Figure 1A in this Norwegian study https://doi.org/10.1186/s12889-024-17695-8 ; Figure 2B in Swedish study https://www.mdpi.com/1999-4915/14/8/1840 and Figure 1B in Danish study https://doi.org/10.1186/s13073-022-01045-7.

Q3.2. Furthermore, enhancing the work by including comparisons to other models or data from different countries could enrich the discussion and conclusions.

R3.2. Collecting the border traffic data for the four modelled countries turned out to be a significant effort. While applying the metrics, presented in this paper, to other countries is possible (they are quite generic), this lies outside the scope of the current project.

Response to Reviewer #4

We thank Reviewer #4 for a thorough reading of our manuscript and providing sharp comments and remarks which have helped to improve the text.

Major comments

Q4.1. L115-121: The text around Gaussian mixture models here is quite confusing. It would be helpful if the authors can better clarify their approach, perhaps with an example.

R4.1. We have clarified the GMM in Appendix S1. We also renamed Appendix S1 into 'Mobility data and model' and added an extra reference to in from the Section 2.2.

Q4.2. Why did they choose to cluster commuters into those staying less or more than a day? This makes some intuitive sense, but since the minimal effect of short term commuters is referenced a couple of times in the manuscript, it would be really helpful to have a clearer rationale for why this division was chosen.

R4.2. The reason behind this particular choice is unavailability of data. While we can estimate the number of commuters (people spending less when a day in the destination country) we cannot distinguish between people spending one, two or ten days abroad. Thus, we had to cluster all non-commuters into a single group. For the sake of simplicity, we assumed that "foreign" infectious individuals' infections recover at the same rate as "local" ones, which allow us to unite them into a single compartment.
More complex models may assume "foreign" infectious individuals to leave the infectious compartment faster - since they already spend a part of their infectious period in the origin country, and since they have some probability to return back. Assuming that would mean that the effect of cross-border mobility is even lower than our estimates. But, since exactly how much faster this happens is impossible to infer from the available data, we stick to the simple model which provides us with the upper bound estimates.
In the discussion, we emphasize that knowing what people do abroad (e.g. how much time they spend there) is critical for planning border-crossing interventions.

Q4.3. Fig1: It would be enormously beneficial to add a third panel here, a graphical methods, or some other schematic summarising the reported parameters in subsequent figures (M, Q, etc). A graphical methods summarising the model and counterfactuals would also be very helpful. I leave this as optional to the authors, but some kind of visual summary here would broaden the potential readership significantly.

R4.3. We added a third panel to Figure 1, schematically showing long-duration travellers and commuters.

Q4.4. L173-178: Other than 50,000 warm-up iterations, did you assess convergence in your posterior?

R4.4. We assessed convergence visually, by looking at the trace plots and comparing quantifies from the different parts of the chain. We attach some of the convergence plots as a separate file for the reviewer.

Q4.5. L192-194: In L129 you state that the modelling period is 2020-02-10 to 2020-12-31, but here you state that estimates are only reported post-April due to unreliable inflow data. Did you use any data prior to April and/or how do you expect it might bias your results? What exactly do you mean by unreliable – is it patchy or biased?

R4.5. To estimate the i^{inflow}, the number of infected individuals arriving from the non-modelled countries, we had to rely on the number of observed cases in these countries. However, for February and March this data is very unreliable (for different reasons and to different degrees in different countries) and thus i^{inflow} cannot be estimated reliably. Consequently the role of imported cases (and its relationship to local transmission or cross-border transmission between Nordic countries) cannot be estimated as well.
The R_t are estimated using the numbers of hospitalizations from the modelled countries, data which we believe to be very reliable.

Q4.6. I am concerned since R_t are auto-correlated a priori (i.e. $R_{t,x} \sim N(R_{t-7,x}, 0.25)$), perhaps unreliable data for inflow may skew estimates and bias inference for later time period?

R4.6. While R_t are indeed correlated a-priori, the posterior correlation is very low.
We added a figure to the appendix S3 showing the posterior correlation of R_t: Figures 12 and 13.

Q4.7. L237: (Eqn 18 and 19): Please keep the superscripts consistent throughout the manuscript. I think π^{ltt} should be π^{long}?

R4.7. Indeed, this is a typo. We replaced π^{ltt} with π^{long}.

Q4.8. L265: I understand what you mean by "net beneficiary" for Sweden in the sense of being able to shed infections elsewhere, but I strongly suggest altering this language since it frames things as a zero-sum game that media may exploit. Perhaps "net exporter" is a better term, and similar language is already used at another point in the manuscript.

R4.8. We agree with this sentiment. We replaced "net beneficiary" with "net exporter".

Q4.9. L268-271: I like how the authors have synthesised their results here. This is a valuable and sorely needed appraisal of the effects of border policy and has practical implications. Please restate this more clearly in the introduction and abstract.

R4.9. We added the following sentence to the introduction and abstract: *Our results indicate that the effect of inter-country mobility on epidemic growth is non-negligible essentially when there is sizeable mobility from a high prevalence country or countries to a low prevalence one.*

Minor comments

L8: Consider swapping SARS and Covid-19 to SARS-CoV-1 and SARS-CoV-2.

We decided keeping using SARS and Covid-19 for brevity.

L9: For relevant genomic studies, please also consider citing `https://doi.org/10.1093/ofid/ofab665`. This article presents genomic evidence supporting the part of the general conclusion here that within-country control measures often exceed effects of travel, connectivity and border policy.

Citation added.

L47: e.g. → "for example"

Corrected.

Fig 2 Caption: Delete "used" in "… and data for hospitalisation and mobility used."

Corrected.

L175: Missing "and". i.e. "… D^{com}, D^{long}, and M^{long} …"

Corrected.

L248: Delete "had experienced" → "experienced"

Corrected.

L250: Reword, and or delete "already"

Corrected.

L307: Perhaps say "June-August" to avoid those of us in the southern hemisphere!

Corrected.

L318: "hundred" → "one-hundred"

Corrected.

L337-342: Please break this up into two sentence because it contains important detail, but is difficult to read as is.

Corrected.

L416: spend → spent

Corrected.

L437: Modeeling

Corrected.

The effect of cross-border mobility on the spread of COVID-19 in Nordic countries.
Appendix S4: Numerical convergence of the Monte Carlo algorithm

March 22, 2024

Mikhail Shubin[1 *], Hilde Kjelgaard Brustad[2], Jørgen Eriksson Midtbø[3], Felix Günther[4], Laura Alessandretti[5], Tapio Ala-Nissila[6, 7], Gianpaolo Scalia Tomba[4, 8], Mikko Kivelä[9], Louis Yat Hin Chan[3], Lasse Leskelä[1]

1 Department of Mathematics and Systems Analysis, Aalto University, Espoo, Finland
2 Oslo Center for Biostatistics and Epidemiology, Oslo University Hospital, Norway
3 Department of Method Development and Analytics, Norwegian Institute of Public Health, Oslo, Norway
4 Department of Mathematics, Stockholm University, Sweden
5 DTU Compute, Technical University of Denmark, Copenhagen, Denmark
6 Quantum Technology Finland Center of Excellence, Department of Applied Physics, Aalto University
7 Interdisciplinary Centre for Mathematical Modelling and Department of Mathematical Sciences, Loughborough University, United Kingdom
8 Department of Mathematics, University of Rome Tor Vergata, Italy
9 Department of Computer Science, Aalto University, Espoo, Finland

* mikhail.shubin@aalto.fi

In this appendix S4 we present MCMC convergence summary.
We check convergence visually, by studying trace plots, quantiles and variance of different parts of the chain of samples.

1 Warm-up iterations

Here we present the trace plots from the first 15,000 warm-up iterations, showing that the distribution were not stable during the initial iterations.

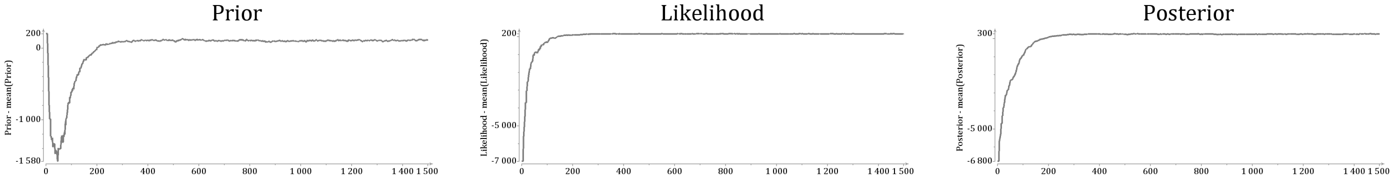

Figure 1: Traceplot for the log prior, log likelihood and log posterior during the warm-up iterations, measured from the means of these values.

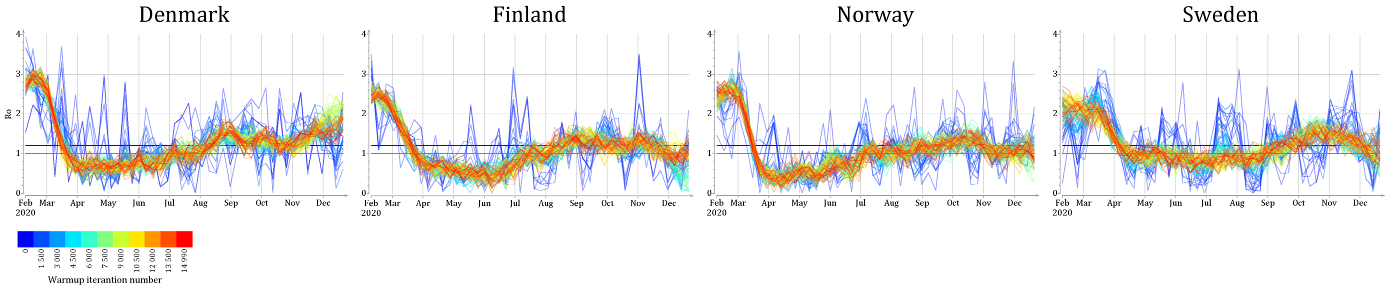

Figure 2: Traceplot for $\mathcal{R}_t$, warmup iterations. 100 samples from $\mathcal{R}_t$ are shown, color represent the iteration's number.

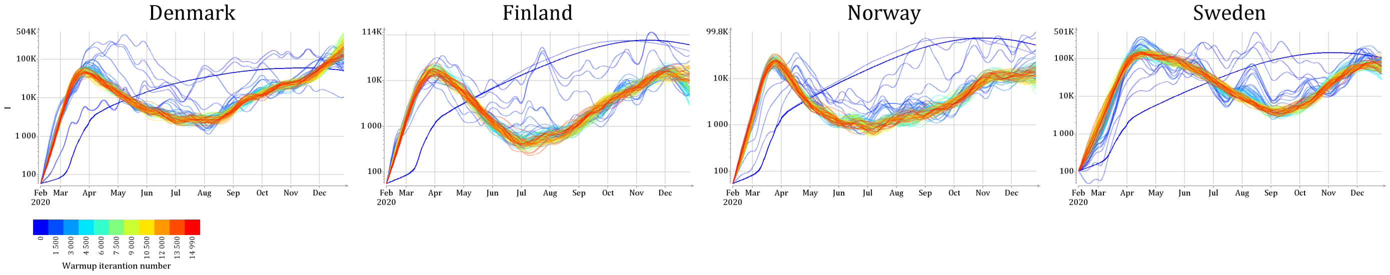

Figure 3: Traceplot for the number of infectious individuals I, warmup iterations. 100 samples from I are shown, color represent the iteration's number.

2 Main iterations

Here we present the trace plots and other metrics for the main 300,000 iterations, showing that the distribution were stable.

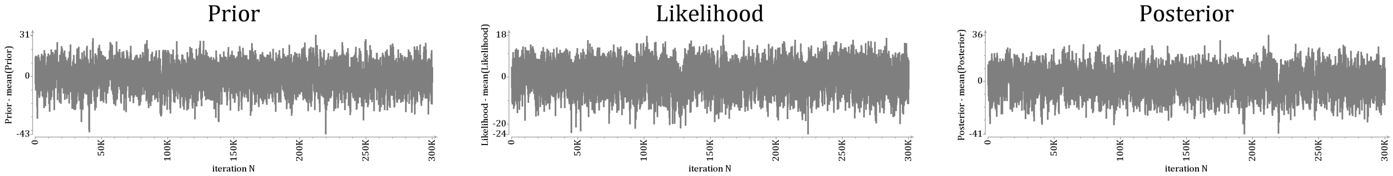

Figure 4: Traceplot for the log prior, log likelihood and log posterior during the main iterations, measured from the means of these values.

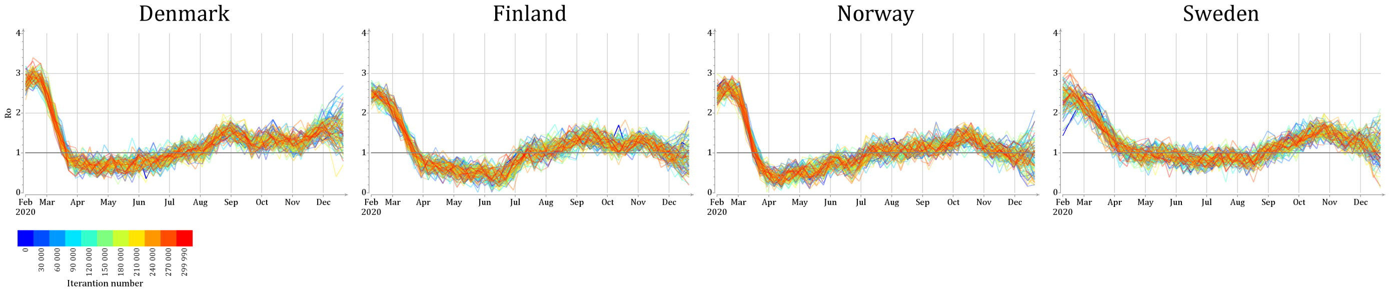

Figure 5: Traceplot for $\mathcal{R}_t$, main iterations. 100 samples from $\mathcal{R}_t$ are shown, color represent the iteration's number.

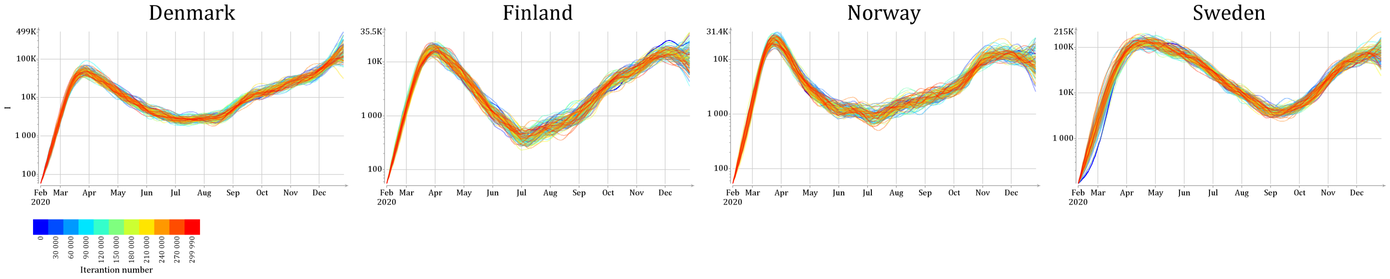

Figure 6: Traceplot for the number of infectious individuals I, main iterations. 100 samples from I are shown, color represent the iteration's number.

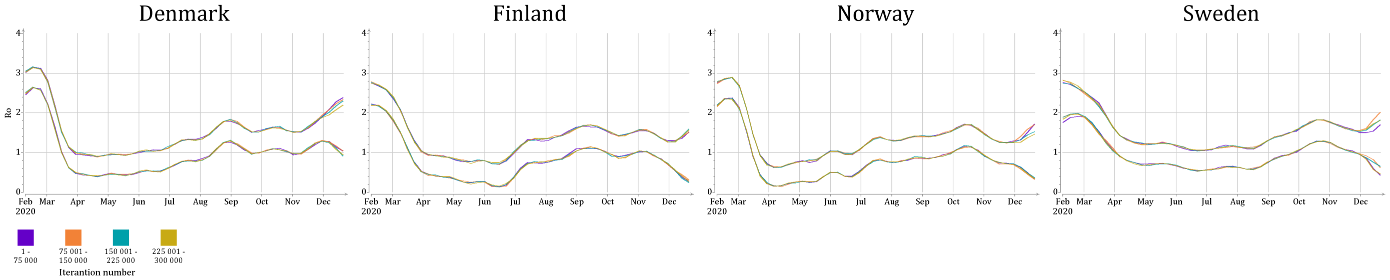

Figure 7: 0.05 and 0.95 posterior quantiles of the $\mathcal{R}_t$, taken separately from the four quarters of a MCMC chain.

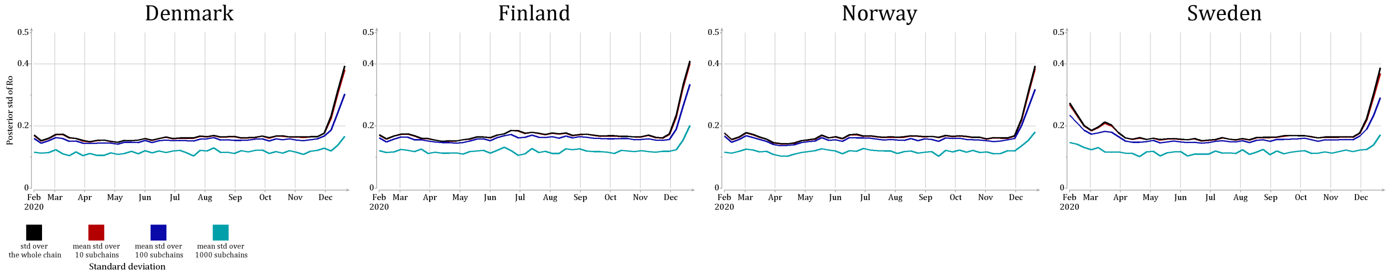

Figure 8: Black lines: posterior standard deviations of $\mathcal{R}_t$ measured on all samples ($std\ \mathcal{R}_t[0:300000]$). Mean posterior standard deviations of $\mathcal{R}_t$ measured on 10 sub-chains ($E(std\ \mathcal{R}_t[i:i+30000])$ for $i = 0, 30000 \ldots 270000$). Mean posterior standard deviations of R_t measured on 100 sub-chains ($E(std\ \mathcal{R}_t[i:i+3000])$ for $i = 0, 3000 \ldots 297000$). Mean posterior standard deviations of R_t measured on 1000 sub-chains ($E(std\ \mathcal{R}_t[i:i+300])$ for $i = 0, 300 \ldots 299700$).

3 Comparison between chains

In addition, we run the Chain additional three times, starting with a random initial parameter values.

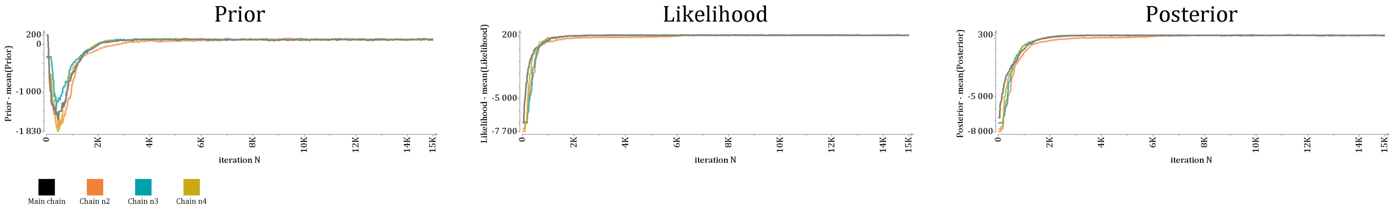

Figure 9: Traceplot for the log prior, log likelihood and log posterior during the warm-up iterations in the main (reported) and additional chains.

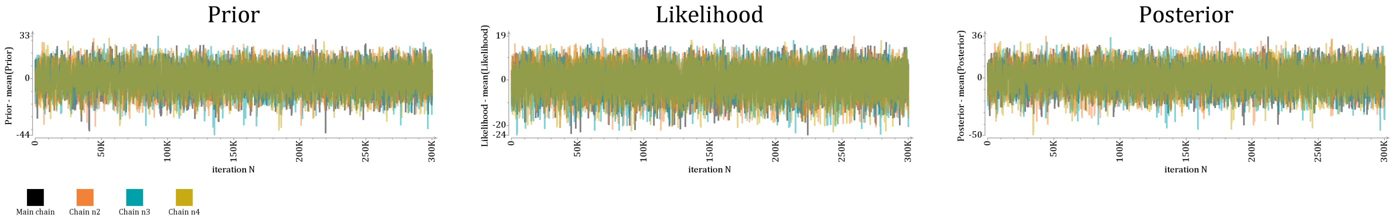

Figure 10: Traceplot for the log prior, log likelihood and log posterior during the main iterations in the main (reported) and additional chains.

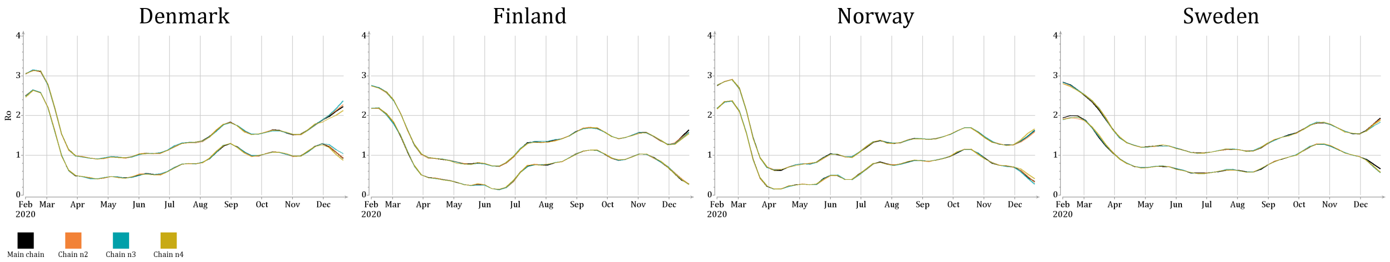

Figure 11: 0.05 and 0.95 posterior quantiles of the $\mathcal{R}_t$, taken separately from the main (reported) chain and three extra chains.
